# Meeting the demands of a podiatry service for patients with arthritis

**DOI:** 10.1186/1757-1146-6-S1-O32

**Published:** 2013-05-31

**Authors:** Keith Rome, Kathryn Erikson, Anthony Ng, Peter J Gow, Hazra Sahid, Anita E Williams

**Affiliations:** 1 Health and Rehabilitation Research Centre, AUT University, Auckland, New Zealand; 2 Department of Rheumatology, Counties Manukau District Health Board, Auckland, New Zealand; 3 University of Salford, Directorate of Prosthetics, Orthotics and Podiatry, Salford, UK

## Background

Despite evidence for the need of podiatry services, podiatry is frequently an underused and under-resourced service and in many areas in New Zealand there is no specialist podiatry service. In support of specialist foot care, a new podiatric rheumatology service was established following an evidence-based approach highlighting the need for improved access to podiatry care for rheumatology patients in New Zealand.

## Methods

A retrospective study of 245 patients with rheumatic disease at Counties Manukau DHB was conducted. Foot pain, impairment and disability were measured using a self-reporting patient outcome measure, the Foot Function Index. A range of podiatric interventions were reported. A self-administered, postal patient satisfaction questionnaire was sent to 148 patients.

## Results

Over two-thirds of patients were observed with hallux valgus (bunions). The results demonstrate a significant reduction in foot pain (p<0.001) from initial visit to second visit (18% reduction in pain). A significant decrease in foot disability (p = 0.04) was found from initial visit to second visit. No significant differences were seen with foot impairment (p=0.78). A variety of intervention measures were used with 24% of patients being prescribed foot orthoses and 28% of patients given footwear advice. The patient satisfaction survey found 84% of patients reported they were satisfied with the new service and 80% of patients reported that the service helped with their foot problems.

## Conclusions

The current service meets the needs of patients who suffer from rheumatological foot conditions such as rheumatoid arthritis and gout. The need for good foot education, provision of foot orthoses and advice on footwear are crucial to reduce the burden on patients with rheumatological foot conditions.

